# Supplementary material for: Returning Individual Tap Water Testing Results to Research Study Participants after a Wildfire Disaster
Source: Int J Environ Res Public Health. 2022 Jan 14;19(2):907. doi: 10.3390/ijerph19020907 (PMC8775780; doi:10.3390/ijerph19020907)
Supplement: Supplementary file 1 [file ijerph-19-00907-s001.zip › ijerph-1523850-supplementary.pdf]

Supplemental Materials

Supplemental Figure S1. Examples of chemical factsheets.

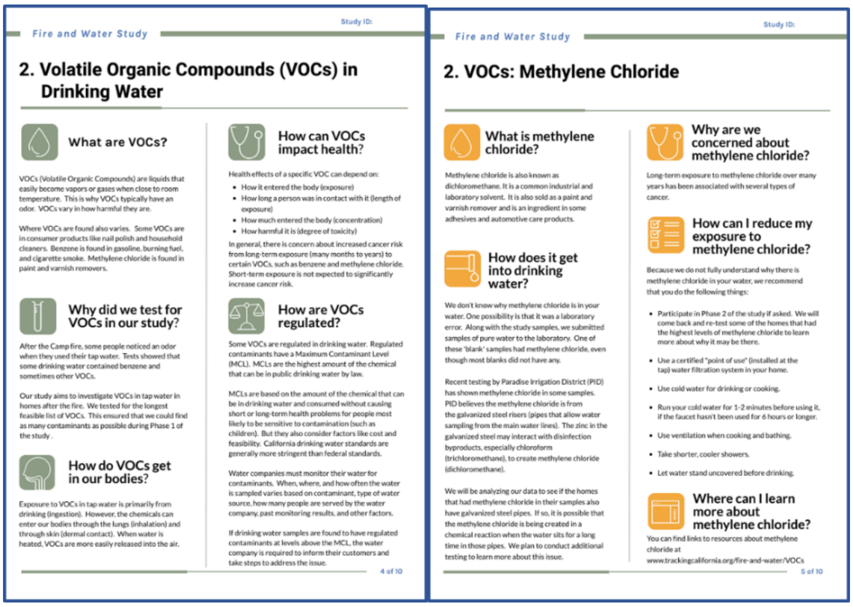

Fire and Water Study

Study ID:

2. Volatile Organic Compounds (VOCs) in Drinking Water

What are VOCs?

VOCs (Volatile Organic Compounds) are liquids that easily become vapors or gases when close to room temperature. This is why VOCs typically have an odor. VOCs vary in how harmful they are.

Where VOCs are found also varies. Some VOCs are in consumer products like nail polish and household cleaners. Benzene is found in gasoline, burning fuel, and cigarette smoke. Methylene chloride is found in paint and varnish removers.

Why did we test for VOCs in our study?

After the Camp fire, some people noticed an odor when they used their tap water. Tests showed that some drinking water contained benzene and sometimes other VOCs.

Our study aims to investigate VOCs in tap water in homes after the fire. We tested for the longest feasible list of VOCs. This ensured that we could find as many contaminants as possible during Phase 1 of the study .

How do VOCs get in our bodies?

Exposure to VOCs in tap water is primarily from drinking (ingestion). However, the chemicals can enter our bodies through the lungs (inhalation) and through skin (dermal contact). When water is heated, VOCs are more easily released into the air.

How can VOCs impact health?

Health effects of a specific VOC can depend on:

- How it entered the body (exposure)
- How long a person was in contact with it (length of exposure)
- How much entered the body (concentration)
- How harmful it is (degree of toxicity)

In general, there is concern about increased cancer risk from long-term exposure (many months to years) to certain VOCs, such as benzene and methylene chloride. Short-term exposure is not expected to significantly increase cancer risk.

How are VOCs regulated?

Some VOCs are regulated in drinking water. Regulated contaminants have a Maximum Contaminant Level (MCL). MCLs are the highest amount of the chemical that can be in public drinking water by law.

MCLs are based on the amount of the chemical that can be in drinking water and consumed without causing short or long-term health problems for people most likely to be sensitive to contamination (such as children). But they also consider factors like cost and feasibility. California drinking water standards are generally more stringent than federal standards.

Water companies must monitor their water for contaminants. When, where, and how often the water is sampled varies based on contaminant, type of water source, how many people are served by the water company, past monitoring results, and other factors.

If drinking water samples are found to have regulated contaminants at levels above the MCL, the water company is required to inform their customers and take steps to address the issue.

4 of 10

Fire and Water Study

Study ID:

2. VOCs: Methylene Chloride

What is methylene chloride?

Methylene chloride is also known as dichloromethane. It is a common industrial and laboratory solvent. It is also sold as a paint and varnish remover and is an ingredient in some adhesives and automotive care products.

How does it get into drinking water?

We don't know why methylene chloride is in your water. One possibility is that it was a laboratory error. Along with the study samples, we submitted samples of pure water to the laboratory. One of these 'blank' samples had methylene chloride, even though most blanks did not have any.

Recent testing by Paradise Irrigation District (PID) has shown methylene chloride in some samples. PID believes the methylene chloride is from the galvanized steel risers (pipes that allow water sampling from the main water lines). The zinc in the galvanized steel may interact with disinfection byproducts, especially chloroform (trichloromethane), to create methylene chloride (dichloromethane).

We will be analyzing our data to see if the homes that had methylene chloride in their samples also have galvanized steel pipes. If so, it is possible that the methylene chloride is being created in a chemical reaction when the water sits for a long time in those pipes. We plan to conduct additional testing to learn more about this issue.

Why are we concerned about methylene chloride?

Long-term exposure to methylene chloride over many years has been associated with several types of cancer.

How can I reduce my exposure to methylene chloride?

Because we do not fully understand why there is methylene chloride in your water, we recommend that you do the following things:

- Participate in Phase 2 of the study if asked. We will come back and re-test some of the homes that had the highest levels of methylene chloride to learn more about why it may be there.
- Use a certified "point of use" (installed at the tap) water filtration system in your home.
- Use cold water for drinking or cooking.
- Run your cold water for 1-2 minutes before using it, if the faucet hasn't been used for 6 hours or longer.
- Use ventilation when cooking and bathing.
- Take shorter, cooler showers.
- Let water stand uncovered before drinking.

Where can I learn more about methylene chloride?

You can find links to resources about methylene chloride at www.trackingcalifornia.org/fire-and-water/VOCs

5 of 10

Supplemental Table S1. Participant responses about the results return communications and the study, Paradise California.

Survey questions	Participants N=90
What communication methods helped you to understand the results? (could choose more than one)	
Written results packets	69 (77%)
Community meeting presentation	19 (21%)
One-on-one contact with Principal Investigator	15 (17%)
Communication with other study staff	11 (12%)
Email of results	1 (1%)
None of these	5 (6%)
No answer	4 (4%)
What aspects of the study were useful to you? (could choose more than one)	
Your water sample results	78 (87%)
Background information about chemicals	49 (54%)
Other information in the packet	32 (36%)
Ability to participate in research project	23 (26%)
Overall study results for the community	14 (16%)
None of these	4 (4%)
No answer	3 (3%)
What inspired your confidence in this study? (could choose more than one)	
Affiliation of project team	66 (73%)
Funder (National Institute of Environmental Health Sciences)	27 (30%)
Study personnel	27 (30%)
Amount or type of information provided	24 (27%)
Study design	23 (26%)
Labs	9 (10%)
None of these	6 (7%)
No Answer	4 (4%)
